# Association between meeting sleep guidelines and anxiety symptoms among Physician assistants in Ghana: a cross-sectional study

**DOI:** 10.3389/frsle.2026.1928518

**Published:** 2026-09-16

**Authors:** Richard Armah Danyi, Patrick Kwame Akwaboah

**Affiliations:** 1 School of Health and Life Sciences, Teesside University, Middlesbrough, United Kingdom; 2 Faculty of Health Sciences, University of Lethbridge, Lethbridge, AB, Canada

**Keywords:** anxiety, Ghana, mental health, physician assistants, sleep duration

## Abstract

**Background:**

Sleep is an important determinant of mental health, yet evidence among healthcare professionals in sub-Saharan Africa remains limited. This study examined the association between adherence to recommended sleep duration guidelines and anxiety symptoms among Physician Assistants in Ghana.

**Methods:**

We conducted a secondary analysis of primary data collected from 439 Physician Assistants in Ghana between October and December 2024. Anxiety symptoms were assessed using the Hospital Anxiety and Depression Scale (HADS), with scores ≥8 indicating anxiety symptoms. Sleep duration was self-reported and categorized as meeting (7–9 h/night) or not meeting (< 7 h/night) recommended sleep guidelines. Multivariable logistic regression was used to examine associations while adjusting for sociodemographic, behavioral, environmental, and climatic factors.

**Results:**

Overall, 31.9% of participants met the recommended sleep duration guideline, while 23.5% reported anxiety symptoms. In adjusted analyses, meeting the recommended sleep guidelines (aOR = 0.45, 95% *CI*: 0.26–0.78) was associated with lower odds of anxiety symptoms. This was confirmed in a sensitivity analysis of sleep duration (hours/day) and anxiety symptoms scores (β = −0.69, 95% CI: −1.01, −0.38). Conversely, practicing in urban settings was associated with higher odds of anxiety symptoms (aOR = 2.17, 95% CI: 1.13–4.16).

**Conclusions:**

Adequate sleep may be an important modifiable factor associated with mental health among Physician Assistants in Ghana. Efforts to support healthy sleep behaviors and create supportive environments may contribute to improved mental health among this population. Future research should include diverse cadres of healthcare professionals and consider a 24-h movement behavior approach.

## 1 Introduction

Anxiety disorders are among the most prevalent mental health conditions globally, affecting approximately 4.4% of the population, with an estimated 359 million individuals affected in 2021 [Global Burden of Disease (GBD), 2024]. Among healthcare professionals, the burden is substantial, with more than half reporting clinically significant anxiety symptoms (COVID-19 Mental Disorders Collaborators, 2021). Anxiety symptoms among healthcare professionals have been associated with reduced wellbeing, impaired job performance, and poorer quality of patient care (Søvold et al., 2021; Bayram Deger, 2024). Identifying modifiable factors associated with anxiety symptoms among healthcare professionals remains a public health priority.

Sleep is a fundamental component of human health and daily functioning. It is essential for cognitive function, emotional regulation, metabolic health, and overall wellbeing (Hirshkowitz et al., 2015). For optimal health, adults aged 18 years and above are recommended to obtain 7 to 9 h of sleep per night, with durations below 7 h considered insufficient (Hirshkowitz et al., 2015; Watson et al., 2015). Insufficient sleep has been associated with several adverse health outcomes, including cardiovascular disease, obesity, and mental health disorders (Irwin, 2015). Experimental and neurobiological evidence further suggests that sleep deprivation is associated with increased amygdala reactivity and impaired prefrontal cortical regulation, mechanisms that have been linked to anxiety symptoms (Goldstein and Walker, 2014; Dressle and Riemann, 2023).

Epidemiological studies consistently report an association between shorter sleep duration and increased anxiety symptoms. In a large analysis of 13,476 adults from the National Health and Nutrition Examination Survey (NHANES), very short sleep duration (< 5 h) was associated with a 40% higher risk of anxiety symptoms (IRR = 1.40, 95% CI: 1.23–1.59), while short sleep duration (5 to < 7 h) was associated with a 17% higher risk (IRR = 1.17, 95% CI: 1.09–1.25), compared with normal sleep duration (7–9 h), after adjustment for confounders (Wang et al., 2025). Similarly, a meta-analysis reported that approximately 47% of healthcare professionals experience insufficient sleep and are more than twice as likely to report anxiety symptoms (Marvaldi et al., 2021). However, much of the available evidence originates from high-income countries. This limits our understanding of how this observed association is in low- and middle-income settings, where healthcare systems, workloads, and working conditions differ substantially.

In Ghana, Physician Assistants serve as key front-line healthcare providers across municipal and district hospitals, health centers, and clinics, often operating in resource-constrained environments with high patient loads (Bawontuo et al., 2021). As of 2021, over 3,000 Physician Assistants were registered with the Medical and Dental Council of Ghana, contributing significantly to outpatient and inpatient care nationwide (Medical and Dental Council of Ghana, 2020). Despite their critical role within the health system, little is known about sleep behaviors and mental health among this professional group.

To our knowledge, no study has examined the association between meeting recommended sleep guidelines and anxiety symptoms among Physician Assistants in Ghana or similar sub-Saharan African settings. Addressing this gap is important for informing occupational health strategies and workforce support policies. Therefore, this study aimed to examine the association between meeting recommended sleep guidelines and anxiety symptoms among Physician Assistants in Ghana. The findings will provide important baseline evidence for future studies involving broader healthcare professional populations and may contribute to occupational health policy discussions in similar settings.

## 2 Methods

### 2.1 Study design and participants

This study followed the Strengthening the Reporting of Observational Studies in Epidemiology (STROBE) guidelines (Vandenbroucke et al., 2007). We used data from a survey conducted among Physician Assistants in Ghana, whose methods has been published elsewhere (Akwaboah et al., 2025). For the primary survey, invitations containing the study link were sent via email to members of the Ghana Physician Assistant Association (GPAA). Additionally, the official GPAA WhatsApp platform was used to distribute the study link. To enhance participation, posters with scannable QR codes were placed at key locations during the annual GPAA conference in October 2024. To ensure data integrity, submissions were restricted to one per email, preventing multiple responses from the same participant. A pre-test was conducted among 35 Physician Assistant interns, and the survey was refined based on their feedback before full implementation. In total, 439 Physician Assistants were recruited between October and December 2024. Ethical approval was obtained from the Metropolitan Research and Education Bureau (MREB) Office of the Research Ethics Review Committee (RERC) under protocol number MREB/RERC/19/24, and electronic informed consent was secured from all participants. All 439 responses were included in the present analysis.

### 2.2 Measures

#### 2.2.1 Outcome variable

Anxiety symptoms were the primary outcome variable for this study, measured using the Hospital Anxiety and Depression Scale (HADS) anxiety subscale. This subscale has demonstrated 80% sensitivity and specificity in previous studies (Bjelland et al., 2002; Vodermaier and Millman, 2011). It consists of seven questions, each rated on a scale from 0 to 3, yielding a total score ranging from 0 to 21. For this study, anxiety symptoms were defined as scores ≥8, a threshold known for its good specificity and sensitivity in a previous study (Mc Dowell et al., 2018). We calculated the internal consistency for this subscale, which demonstrated very good reliability (Cronbach's alpha = 0.84).

#### 2.2.2 Exposure

Sleep duration (hours/day) was self-reported by participants. It was assessed with the question:

“On average, how many hours of sleep do you get per night? (state the number, e.g., 5) ——— hours.”

For this study, we categorized sleep based on the Canadian 24-h Movement Guidelines for adults (Ross et al., 2020). Participants reporting 7–9 h of sleep per night were classified as meeting the recommended sleep guideline, while those reporting less than 7 h were classified as not meeting the guideline. No participant reported sleep durations exceeding 9 h in the primary survey.

#### 2.2.3 Covariates

Covariates (i.e., age, sex, educational attainment, monthly income, relationship, alcohol intake, and physical activity) were selected a priori based on their associations with sleep duration and anxiety symptoms (Zhang et al., 2024). Additionally, sidewalk availability, which is representative of walkability (Yoon and Oh, 2026) and climatic factors (Rifkin et al., 2018) were also adjusted for based on their observed associations with sleep.

Sociodemographic variables included age, sex, educational attainment, monthly income, and relationship. Age was self-reported and later categorized into two groups (24–40 years and 41–67 years). Sex was reported as male or female. Educational attainment was recorded as advanced diploma, bachelor's degree, and master's and above. Monthly income was categorized as ≥ Gh¢5,000 and < Gh¢5,000, while marital status was classified as married or not married.

Behavioral factors included alcohol intake (yes/no) and moderate-to-vigorous physical activity (MVPA). MVPA was assessed using a modified version of the Global Physical Activity Questionnaire (GPAQ), which has been validated for assessing PA levels (Cleland et al., 2014). Weekly MVPA minutes were calculated based on the number of days and duration of reported activities. MVPA was subsequently categorized as sufficient (≥150 min per week) or insufficient (< 150 min per week) in accordance with World Health Organization recommendations (World Health Organization, 2020).

Environmental variables included practice location (urban/rural) and availability of sidewalks along the route to work (yes/no). Each participant also reported the region in which they practiced, selecting from Ghana's 10 administrative regions. Monthly data on climatic factors (rainfall, temperature, relative humidity) for the period October to December 2024, derived from satellite-based monitoring stations located within each region, were obtained from the Ghana Meteorological Agency. For analysis, weather variables were averaged across the 3-month period for each region and linked to participants based on their reported practice location.

### 2.3 Statistical analysis

Descriptive statistics were used to summarize participant characteristics. Continuous variables were reported as means and standard deviations, while categorical variables were presented as frequencies and percentages.

Bivariate analyses were conducted to examine differences in meeting sleep guidelines and anxiety symptoms across sex and age groups. Pearson's chi-square test was used to assess these associations, with statistical significance evaluated at *p* < 0.05.

To examine the association between meeting recommended sleep guidelines (7–9 h) and experiencing anxiety symptoms, multivariable logistic regression models were fitted. Anxiety symptoms were treated as a binary outcome variable, and results were reported as adjusted odds ratios with corresponding 95% confidence intervals. The model adjusted for relevant covariates, including sociodemographic, behavioral, and selected environmental and climatic factors. As a sensitivity analysis, a linear regression model treating both sleep and anxiety symptom as continuous variables was conducted.

Model fit was assessed using the Hosmer–Lemeshow goodness-of-fit test, which indicated an adequate fit (*p* = 0.5057). Additionally, there was no issues of multicollinearity (VIF < 3). All analyses were conducted using Stata (version 19.0). Statistical significance was set at *p* < 0.05 for all inferential analyses.

## 3 Results

The majority of participants were male (65.8%), held a bachelor's degree (69.5%), and practiced in urban settings (78.8%) (Table 1).

**Table 1:** Participant characteristics.

Variables	Overall (*n* = 439)
	**Mean** ±**SD/n (%)**
Rainfall (mm)	109.9 ± 69.8
Temperature (°C)	28.2 ± 0.7
Relative Humidity (%)	74.3 ± 11.0
**Age Group**	
24–40 years	324 (73.8)
41–67 years	115 (26.2)
Sex	
Female	150 (34.2)
Male	289 (65.8)
Education level	
Advanced diploma	42 (9.6)
Bachelors	305 (69.5)
Master's and above	92 (21.0)
Income (monthly)	
Gh¢5,000 and above	231 (52.6)
Below Gh¢5,000	208 (47.4)
Relationship	
Married	334 (76.1)
Not married	105 (23.9)
Alcohol intake	
No	364 (82.9)
Yes	75 (17.1)
Practice location	
Rural	93 (21.2)
Urban	346 (78.8)
Availability of side walks	
No	292 (66.5)
Yes	147 (33.5)
MVPA guidelines	
< 150 min per week	304 (69.3)
≥150 min per week	135 (30.7)

Table 2 presents sex and age group differences in sleep guideline adherence and anxiety symptoms. Overall, 31.9% of participants met the recommended sleep duration of 7–9 h. A higher proportion of females met the sleep guideline compared to males (39.3% vs. 28.0%, *p* = 0.016). The overall prevalence of anxiety symptoms was 23.5%, with a higher proportion observed among the younger age group compared to the older age group (26.2% vs. 15.7%, *p* = 0.021).

**Table 2:** Sex and age-group differences in sleep guidelines and anxiety symptoms.

	Sex Differences (%)				Age Differences (%)		
**Variable**	**Overall (*****N*** = **439)**	**Male (*****N*** = **289)**	**Female (*****N*** = **150)**	***P***	**24–40 yrs (*****N*** = **324)**	**41-67 yrs (*****N*** = **115)**	***P***
**Sleep guidelines**							
< 7 h	68.1	72.0	60.7	**0.016**	69.1	65.2	0.439
7–9 h	31.9	28.0	39.3		30.9	34.8	
**Anxiety symptoms**							
No	76.5	75.1	79.3	0.319	73.8	84.3	**0.021**
Yes	23.5	24.9	20.7		26.2	15.7	

Bold values represent statistically significant results.

The association between meeting sleep guidelines and anxiety symptoms is presented in Table 3. The overall model had a McFadden pseudo *R*^2^ of explained 0.078. In the multivariable logistic regression model, meeting the recommended sleep guideline was significantly associated with lower odds of anxiety symptoms (aOR = 0.45, 95% CI: 0.26–0.78). Participants practicing in urban settings had significantly higher odds of anxiety symptoms (aOR = 2.17, 95% CI: 1.13–4.16). In contrast, while the availability of sidewalks along the route to work was associated with lower odds of anxiety symptoms (aOR = 0.59, 95% CI: 0.35–0.99), the findings should be interpreted with caution the confidence interval is close to the null.

**Table 3:** Association between sleep guidelines and anxiety symptoms.

**Variable**	aOR	95% *CI*
**Sleep guidelines**		
< 7 h	REF	REF
7–9 h	**0.45** ^**^	**0.26–0.78**
Temperature (°C)	1.50	0.85**–**2.65
Rainfall (mm)	1.00	0.99**–**1.01
Relative humidity (%)	1.00	0.98**–**1.03
**Age group**		
24–40 years	REF	REF
41–67 years	0.58	0.31–1.08
Sex		
Female	REF	REF
Male	1.20	0.70–2.03
Education		
Advanced diploma	REF	REF
Bachelors	2.44	0.87–6.83
Master's and above	2.80	0.92–8.55
Income (monthly)		
≥ ¢5,000	REF	REF
< ¢5,000	1.41	0.85–8.07
Alcohol intake		
No	REF	REF
Yes	1.54	0.86–2.77
Practice location		
Rural	REF	REF
Urban	**2.17** ^*^	**1.13–4.16**
**Availability of side walks**		
No	REF	REF
Yes	**0.59** ^*^	**0.35–0.99**
**Relationship**		
Married	REF	REF
Not married	1.10	0.63–1.93
MVPA guidelines		
< 150 min per week	REF	REF
≥150 min per week	1.58	0.96–2.61

aOR, adjusted odds ratio.

CI, confidence intervals.

REF, reference.

** *P* < 0.01; ^*^P < 0.05.

Bold values represent statistically significant results.

In the sensitivity analysis, each 1-h increase in daily sleep duration was associated with a 0.69-point decrease in anxiety symptom (β = −0.69, 95% CI: −1.01, −0.38), consistent with the primary analysis (Table 4).

**Table 4:** Association between sleep duration and anxiety symptoms scores: sensitivity analysis.

Variable	β	SE	95% CI	Adjusted *R*^2^
Sleep duration (hours/day)	**−0.69** ^***^	**0.16**	**−1.01**, **−0.38**	**0.075**

β, beta coefficient.

SE, standard error.

CI, confidence interval.

*** *P* < 0.001.

Model was adjusted for age, sex, education, relationship, monthly income, alcohol intake, sidewalk availability, moderate-to-vigorous physical activity guidelines, rainfall, temperature, and relative humidity. Bold values represent statistically significant results.

## 4 Discussion

This study examined the association between meeting sleep guidelines and anxiety symptoms among Physician Assistants in Ghana. Overall, 23.5% of respondents reported anxiety symptoms, with a significantly higher prevalence among younger participants aged 24–40 years compared to those aged 41–67 years (26.2% vs. 15.7%, *p* = 0.021). Only 31.9% of participants met the recommended sleep duration of 7–9 h per night, with females more likely than males to meet the guideline (39.3% vs. 28.0%, *p* = 0.016). In adjusted analyses, meeting sleep guidelines and the availability of sidewalks on the route to work were associated with 55% and 41% lower odds of anxiety symptoms, respectively. Conversely, practicing in an urban setting was associated with two-fold higher odds of anxiety symptoms.

The prevalence of anxiety symptoms observed in this study was substantial, although lower than that reported among healthcare workers in Ethiopia (57.6%) (Habtu et al., 2024). Nevertheless, the observed prevalence falls within the range reported across sub-Saharan Africa (Too et al., 2025), underscoring the considerable mental health burden experienced by healthcare professionals in the region. The higher prevalence among younger Physician Assistants may reflect greater occupational stress associated with career establishment, professional uncertainty, and workload demands during the early stages of practice.

The proportion of participants meeting sleep duration recommendations (31.9%) was relatively low compared with estimates reported among Canadian adults (Wang et al., 2022; Rollo et al., 2023), but was comparable to findings from Latin American populations (Ferrari et al., 2022). Females were more likely than males to meet the recommended sleep duration guideline, although the factors underlying this difference warrant further investigation. Overall, variations in sleep guideline adherence across settings may reflect differences in occupational demands, work schedules, socioeconomic conditions, healthcare system structures, study populations, and measurement approaches.

Meeting recommended sleep duration was significantly associated with lower odds of experiencing anxiety symptoms. This was supported by the sensitivity analysis, in each 1-h increase in daily sleep duration was associated with a 0.69-point decrease in anxiety symptoms score. This findings in our study is consistent with a growing body of evidence linking inadequate sleep to poorer mental health outcomes, including anxiety disorders (Wang et al., 2025). Meta-analytic evidence indicates that sleep disturbances are highly prevalent among individuals with anxiety disorders (Baglioni et al., 2016), while interventions that improve sleep quality and duration have been associated with reductions in anxiety symptoms and overall improvements in mental wellbeing (Scott et al., 2021). Although the cross-sectional design precludes causal inference, the findings reinforce the importance of adequate sleep as a potentially modifiable factor associated with psychological wellbeing among healthcare professionals, who play a critical role in maintaining health system performance.

The availability of sidewalks on the route to work was associated with lower odds of experiencing anxiety symptoms, while practicing in an urban setting was associated with higher odds. While they highlight the potential role of the built environment in shaping mental wellbeing among healthcare professionals, the observed association between sidewalk availability should be interpreted cautiously because it is secondary to the main research question and its confidence interval is close to the null. As such, the discussion on these findings is exploratory. Urban environments often expose individuals to stressors such as traffic congestion, noise, and increased work demands, which may contribute to anxiety. Conversely, supportive infrastructure may help mitigate these effects. For example, healthcare workers exposed to green urban environments have been shown to experience lower anxiety levels than those in barren urban settings (Saeidi-Rizi et al., 2025).

### 4.1 Strengths and limitations

This study has several strengths. To our knowledge, it is among the first studies to examine the association between sleep guideline adherence and anxiety symptoms among healthcare professionals in Ghana and the wider West African region. Additionally, anxiety symptoms were assessed using the Hospital Anxiety and Depression Scale, a widely used and validated instrument with good reliability in the present study.

However, several limitations should be acknowledged. First, the cross-sectional design precludes conclusions regarding causality or the temporal direction of the observed associations. Second, sleep duration was self-reported and may be subject to recall and social desirability bias. Third, the study assessed sleep duration but did not capture other important dimensions of sleep, such as sleep quality, insomnia symptoms, or sleep timing. Fourth, reverse causation is imminent: anxiety symptoms may influence sleep duration just as inadequate sleep may be associated with anxiety. Additionally, the results may not be generalizable to the wider healthcare professionals' group. Finally, unmeasured factors, including workload, shift patterns, burnout, and social support, may have influenced the observed associations.

### 4.2 Future directions

Future studies should employ longitudinal designs to better understand the temporal relationship between sleep duration and anxiety symptoms among healthcare professionals. Research incorporating objective measures of sleep, such as actigraphy, alongside assessments of sleep quality and workplace factors, would provide a more comprehensive understanding of the determinants of mental wellbeing. Additionally, future research should consider the integrated 24-h movement behavior framework, examining the combined and independent contributions of sleep, physical activity, and sedentary behavior to mental health outcomes. Such an approach may provide a more holistic understanding of lifestyle factors associated with anxiety among healthcare professionals. Further investigation into built environment characteristics, including sidewalk quality, walkability, and access to green spaces, may also help identify environmental strategies for promoting mental wellbeing in this population.

## 5 Conclusion

Promoting the mental health of Physician Assistants is essential for maintaining a resilient health system. This study highlights the potential importance of adequate sleep as a key modifiable factor of anxiety symptoms among Physician Assistants in Ghana, which can potentially contribute to a healthier and more productive workforce. Future studies should adopt a 24-h movement behavior perspective using objective measures; to better understand how daily movement patterns influence mental health outcomes among a wider healthcare professional populace.

## Data Availability

The data analyzed in this study is subject to the following licenses/restrictions: the data set are only available upon request. Requests to access these datasets should be directed to pk.akwaboah@uleth.ca.
